# Smoking cessation among European older adults: the contributions of marital and employment transitions by gender

**DOI:** 10.1007/s10433-016-0401-4

**Published:** 2016-10-21

**Authors:** Sergi Trias-Llimós, Magdalena M. Muszyńska, Antonio D. Cámara, Fanny Janssen

**Affiliations:** 1 Population Research Centre, Faculty of Spatial Sciences, University of Groningen, Landleven 1, 9747 AD Groningen, The Netherlands; 2 Institute of Statistics and Demography, Warsaw School of Economics, Al. Niepodległości 162, 02-554 Warsaw, Poland; 3 Department of Business Management, Marketing and Sociology, University of Jaén, Campus de las Lagunillas, s/n Edificio D-3 (dep. 007), 23071 Jaén, Spain; 4 Netherlands Interdisciplinary Demographic Institute, Lange Houtstraat 19, 2511 CV The Hague, The Netherlands

**Keywords:** Stressful events, Smoking cessation, Older adults, SHARE, Europe

## Abstract

Knowledge about the potential effects of stressful events on smoking cessation is helpful for the design of health interventions. Previous studies on this topic tended to group together adults of all ages and of both genders. We investigate the contribution of marital and employment losses on smoking cessation by gender, specifically among older adults in Europe. We used panel data from waves 4 (2011) and 5 (2013) of the Survey of Health, Ageing and Retirement in Europe for 3345 male and 3115 female smokers at baseline aged 50 and over from 13 countries. The associations between marital and employment losses and smoking cessation were derived from logistic regression models for each gender, controlling for age, educational attainment, diseases incidence and country of residence. Interactions between gender and marital and employment losses were tested. Over the analysed period, 119 smokers became widowed or divorced (1.8 %), 318 became retired (4.9 %) and 100 became unemployed (1.5 %). Becoming widowed or divorced was associated with lower probability of smoking cessation among both men (OR 0.36, 95 % CI 0.14–0.94) and women (OR 0.46, 95 % CI 0.21–0.99). Transitions to unemployment and to retirement were not significantly associated with smoking cessation (OR 0.62, 95 % CI 0.25–1.49; and OR 0.68, 95 % CI 0.43–1.07, respectively). Gender differences in the association between marital and employment losses and smoking cessation were not statistically significant (*p* value > 0.05 for all interactions). Health interventions should take into account that male and female older adults affected by marital loss are at risk of continuing smoking.

**Electronic supplementary material:**

The online version of this article (doi:10.1007/s10433-016-0401-4) contains supplementary material, which is available to authorized users.

## Introduction

Smoking is the leading risk factor in the burden of disease and mortality in Europe (Lim et al. 2012), and one of the most important public health issues in Europe. Assessing smoking cessation determinants is important because smoking cessation significantly improves people’s health and reduces their mortality risk (Burns 2000; Doll et al. 2004; Taylor et al. 2002). Prior studies have shown that smoking cessation is consistently associated with demographic characteristics, such as sex and age; and that it is influenced by many factors that are amenable to change, including socio-economic factors, social support and the prevalence of smoking-related diseases (e.g. van den Putte et al. 2005; Broms et al. 2004; Margolis 2013; van Gool et al. 2007). Given the potential for changing many determinants of smoking cessation and the benefits of smoking cessation, gaining additional knowledge about the determinants of smoking cessation can aid policy-makers aiming to improve public health by promoting smoking cessation.

Previous studies on the determinants of smoking cessation mainly focused on the role of socio-economic status, social support or psychosocial determinants in smoking cessation (e.g. Osler and Prescott 1998; Janzon et al. 2005), and paid less attention to the associations between key life course transitions and smoking cessation. The studies that investigated the role of key life course transitions found that smoking cessation is associated with both marital transitions (Giordano and Lindstrom 2011; Kriegbaum et al. 2011; Lee et al. 2005; Nystedt 2006) and employment transitions (Blakely et al. 2014; De Vogli and Santinello 2005; Giordano and Lindstrom 2011; Kriegbaum et al. 2011; Lang et al. 2007). These analyses also showed that marital loss and employment loss influence smoking cessation via stress (De Vogli and Santinello 2005; Falba et al. 2005; Johnson and Wu 2002; Thomas et al. 2005), which usually hinders efforts to quit. However, because most of these studies examined all adult age groups combined, little is known about the associations between key life transitions and smoking cessation among older adults. In analysing the determinants of smoking cessation, and specifically the role of marital and employment transitions, it is essential that older adults are studied separately (Jarvis et al. 2013) for a number of reasons. First, although the benefits of smoking cessation are greatest for smokers who quit early in life, quitting later in life is also associated with lower mortality (Doll et al. 2004; Taylor et al. 2002). Second, older adults are of special concern because they tend to need more health care than younger people, and because the number of older people in Europe is growing. Third, older adults are more likely to quit smoking than younger adults (Osler et al. 1999), largely because older adults are more likely than younger adults to suffer from various smoking-related diseases, which is regarded as a motivation to quit smoking (Margolis 2013; van Gool et al. 2007). Fourth, marital and employment loss, particularly widowhood and retirement, are prevalent at older ages. Fifth, the effects of various stressful events on smoking have been shown to differ over the life course (Grotvedt and Stavem 2005; Jarvis et al. 2013; Umberson et al. 2008; Whitson et al. 2006).

Although the importance of studying smoking cessation separately for different age groups has been acknowledged in the literature, the existing evidence regarding the importance of marital and employment losses to smoking cessation at older ages is both scarce and mixed. We are aware of only two studies that specifically examined smoking cessation among middle-aged and older adults in relation to marital transitions: one study that focused on female nurses (Lee et al. 2005), and another that looked at male health professionals (Eng et al. 2005); both in the US. These studies found that becoming widowed and remaining unmarried were associated with lower smoking cessation rates among these older women (Lee et al. 2005), but these transitions were not found to result in significant differences in smoking behaviour among older men (Eng et al. 2005). Although these studies observed some interesting potential gender differences, the narrowness of the selected group and settings affected the generalisability of the results.

In the literature on the association between employment transitions and smoking cessation, there has been mixed evidence regarding two of the main work-related transitions experienced by older adults: retirement and unemployment. For example, a British study found a positive association between retirement and smoking cessation (Lang et al. 2007). However, no such association was found in research conducted in the US (Midanik et al. 1995) or in the Netherlands (Henkens et al. 2008). Being unemployed was found to be negatively associated with smoking cessation in a study among British adults (Giordano and Lindstrom 2011), but the transition from being employed to being unemployed was not shown to be associated with lower chances of smoking cessation in the samples of older British (Falba et al. 2005) or German adults (Schunck and Rogge 2012).

Most of the previous studies on this topic did not distinguish between men and women. Yet the masking of gender differences could affect the overall results (Grotvedt and Stavem 2005). Only a few studies have reported gender-specific results among adults (McKee et al. 2003; Nystedt 2006). On the one hand, gender differences were not found in the association between marital disruption and smoking cessation (Nystedt 2006), or in the association between interpersonal loss and smoking behaviour (McKee et al. 2003). On the other hand, women were found to be less likely than men to quit smoking after financial or health events (McKee et al. 2003).

Various mechanisms are likely to affect the smoking cessation chances of men and women differently. Stress levels linked to marital and employment loss may not be the same for men and women, as men tend to report experiencing more work-related stressful events while women tend to report experiencing more interpersonal stressful events (Kendler et al. 2001). These gender differences may be due to differences between men and women in terms of their role configurations (Ensminger and Celentano 1990), and in their strategies for coping with stressful life course circumstances (Kessler and McLeod 1984). Furthermore, it is also well known that marriage has a more protective effect for men than for women in terms of the adoption of unhealthy behaviours (Schone and Weinick 1998), adverse health outcomes and even mortality (Lillard and Panis 1996). Similarly, the health and mortality outcomes of men have been found to deteriorate more than those of women after widowhood (Moon et al. 2011).

In this study, we seek to add to the existing knowledge on the determinants of smoking cessation by studying the gender-specific associations between marital and employment losses and smoking cessation among older adults (i.e. individuals aged 50 and over) in Europe. We use a longitudinal perspective to examine these transitions over a two-year period (2011–2013).

We hypothesise that potential stressful transitions in life, such as marital and employment losses, could have different effects on the smoking cessation chances of men and women. We also expect that our results for older adults will differ from the findings of previous research that did not focus on this specific age category.

## Materials and methods

### Data

We used panel data on smoking behaviour as well as socio-demographic and health-related variables from wave 4 (2011: baseline) and wave 5 (2013: follow-up) of the Survey of Health, Ageing and Retirement in Europe (SHARE) (Börsch-Supan et al. 2005) for individuals aged 50 and over in the 13 countries covered by the two waves: Austria, Belgium, Czech Republic, Denmark, Estonia, France, Germany, Italy, the Netherlands, Slovenia, Spain, Sweden and Switzerland. We have used the most recent SHARE data, which indeed provided larger sample size than any other pair of SHARE waves. In addition, these waves are only two years apart, which implies that the probability that the respondents experienced more than one marital or employment transition is reduced. However, using both waves provided us with a sample size large enough for the purposes of this study. Of the individuals who participated in both waves (*n* = 50,332, 74.3 % of the individuals at baseline), we selected those who smoked at baseline (20.4 % of the men and 14.1 % of the women). We excluded 110 cases (1.7 %) with missing values in any of the independent variables resulting in a sample of 3345 men and 3115 women.

#### Dependent variable

The dependent variable of this study is smoking cessation over a two-year period (2011–2013). Depending on the answer at the follow-up in 2013 to the question “Do you smoke at the present time?”, smokers in 2011 were classified into either ex-smoker (1) or still smoking (0) in 2013.

#### Independent variables (covariates)

The main covariates in this analysis were marital and employment losses. In addition we have controlled by age, education, incidence of diseases, country of residence and gender (in the combined model).

Marital status originally had six categories. Three categories represented individuals who were in a union: “married and living together with spouse”, “married, living separated from spouse” and “registered partnership”; while the other three categories represented individuals who were not in a union: “never married”, “divorced” and “widowed”. From the outcome of the two waves, three different transition categories were created based on the assumption that no more than one transition had occurred over the analysed period: “stayed in a union”, “became widowed or divorced” (from union to either widowed or divorced) and “not in a union”. We excluded from the analysis cases with transitions in a union due to small sample size (35 cases).

Employment status originally had six categories: “retired”, “employed or self-employed (including working for family business)”, “unemployed and looking for work”, “permanently sick or disabled”, “homemaker” and “other (rentier, living off of property, student, doing voluntary work)”. We re-categorised employment status to account for the transition between waves into: “stayed (self-)employed”, “became retired” (from (self-)employed), “stayed retired”, “from (self-)employed to unemployed”, “sick, disabled, or other unemployed” (which contains individuals who were in any of these states or transiting between them), “stayed homemaker” and a heterogeneous remaining group of “others”.

The age at baseline was estimated using the year and the month of birth and the year and the month of the interview, and was included in the models as continuous variable.

Educational attainment was built by SHARE according to the International Standard Classification of Education (ISCED) 1997. Following previous studies (Avendano et al. 2009), the seven categories of this variable were reclassified in three categories: lower secondary school or lower (groups 0–2), upper secondary school (group 3) and post-secondary school (groups 4–6).

The detection of a smoking-related disease is one of the main reasons why people quit smoking (Margolis 2013; McKee et al. 2003; van Gool et al. 2007). Chronic conditions (hypertension, high cholesterol, diabetes and lung disease) and major smoking-related diseases (heart attack and cancer) were used to control for disease incidence over the analysed period.

Country of residence was included to control for potential heterogeneity in our sample. Apart from economic and cultural differences, which likely affected the occurrence of employment losses, European countries were subject to different tobacco control policies and to potential changes in these policies. These differences may have affected smoking cessation propensities across countries (Lemmens et al. 2008; Schaap et al. 2008).

### Analysis

Multivariate logistic regressions with smoking cessation as the dependent variable and with marital and employment transitions as independent variables were run for men and women; all controlled for age (as a continuous variable). Additional covariates were added one by one (education, disease incidence and country of residence) in models 2–4, respectively (see Supplementary Tables 1, 2). Model 4, which includes all of the covariates, is the final model presented in the results section. In addition we have run the models for both genders combined (for comparison purposes), adding gender as a control variable. To test if marital and employment losses are differently associated with smoking cessation between men and women we have included the interactions between gender and marital transitions, and between gender and employment transitions.

## Results

In our study sample of 6460 smokers (3345 men and 3115 women) aged 50 and over in 2011, 613 men (18.3 %) and 556 women (17.8 %) had quit smoking by 2013. Transitions from marriage were more common among women (70 out of 3115; 2.2 %) than among men (49 out of 3345; 1.5 %). By contrast, men experienced slightly more employment transitions (5.4 % retired and 1.5 % became unemployed) than women (4.5 and 1.6 %, respectively) (Table 1).

**Table 1:** Background characteristics of smokers in 2011 and percentages of smokers who had stopped smoking by 2013. *Source* Own estimations based on waves 4 and 5 of SHARE

	Men (*n* = 3345)		Women (*n* = 3115)		Total (*n* = 6460)	
	Smokers in 2011 (*n*)	% of the smokers who stopped by 2013	Smokers in 2011 (*n*)	% of the smokers who stopped by 2013	Smokers in 2011 (*n*)	% of the smokers who stopped by 2013
Marital transitions						
Stayed in a union	2468	19.9	1762	18.7	4230	19.4
Became widowed or divorced	49	10.2	70	11.4	119	10.9
Not in a union	828	14.0	1283	17.1	2111	15.9
Employment transitions						
Stayed employed	909	16.6	853	15.8	1762	16.2
Became retired	179	15.6	139	16.5	318	16.0
Stayed retired	1532	21.7	1223	19.1	2755	20.6
From employed to unemployed	51	11.8	49	8.2	100	10.0
Sick/disabled or other unemployed	298	10.1	199	16.6	497	12.7
Stayed homemaker	0	–	220	18.2	220	18.2
Others	376	17.3	432	20.1	808	18.8
Age (baseline)						
50–59	1458	13.7	1597	14.9	3055	14.3
60–69	1307	18.5	1061	18.8	2368	18.6
70+	580	29.5	457	26.0	1037	28.0
Education (ISCED-97)						
0–2: Lower secondary school	1272	19.8	1188	17.7	2460	18.8
3: Upper secondary school	1277	14.6	1215	18.4	2492	16.4
4–6: Post-secondary school	796	22.0	712	17.3	1508	19.8
Newly diagnosed diseases						
Hypertension	334	22.8	280	21.4	614	22.1
Cholesterol	289	20.8	274	18.2	563	19.5
Diabetes	114	26.3	99	21.2	213	23.9
Lung disease	133	23.3	132	22.0	265	22.6
Heart attack	170	32.4	91	22.0	261	28.7
Cancer	86	29.1	73	31.5	159	30.2
Country						
Austria	358	17.9	370	14.6	728	16.2
Belgium	324	14.5	327	14.4	651	14.4
Czech Republic	394	16.8	472	15.3	866	15.9
Denmark	204	17.2	200	22.5	404	19.8
Estonia	638	15.7	394	17.3	1032	16.3
France	274	11.7	246	16.7	520	14.0
Germany	81	22.2	73	24.7	154	23.4
Italy	215	27.4	191	29.3	406	28.3
Netherlands	139	22.3	192	15.1	331	18.1
Slovenia	145	15.2	128	9.4	273	12.5
Spain	236	24.6	146	21.9	382	23.6
Sweden	60	41.7	109	30.3	169	34.3
Switzerland	277	20.2	267	18.4	544	19.3

Significant and positive ORs of quitting smoking by age were observed among both genders (*p* value < 0.001) (see Online Supplementary Tables 1, 2). The expected educational gradient in quitting smoking was not found to be significant although the ORs for the highest educational group were larger than the ORs of the reference category: the lowest educated group (men: OR 1.22, 95 % CI 0.94–1.56; women: OR 1.08, 95 % CI 0.82–1.41). The results for disease incidence indicated that all of the newly diagnosed diseases were positively associated with smoking cessation as compared to not having the disease, but only the incidence of the following diseases was statistically significantly associated with higher smoking cessation ORs: hypertension and heart attack for men and cancer for both men and women. For example, the chances of quitting smoking were twice as high for the individuals with cancer incidence relative to the individuals who did not suffer cancer (OR 1.81, 95 % CI 1.10–2.98, and OR 2.06, 95 % CI 1.22–3.46, for men and women, respectively). Finally, relative to the country with lower share of smokers who stopped by 2013 (Poland for men and Slovenia for women) we found that Italy, the Netherlands, Spain and Sweden had statistically higher ORs of smoking cessation for men, and that Denmark, Germany, Spain and Sweden had higher ORs for women.

### The effect of marital losses

Becoming widowed or divorced was negatively associated with smoking cessation compared to remaining in a union for both men (OR 0.36, 95 % CI 0.14–0.94) and women (OR 0.46, 95 % CI 0.21–0.99) (Table 2). The ORs for remaining not in a union were greater but also negatively associated with smoking cessation among men (OR 0.72, 95 % CI 0.57–0.90). This association was not significant among women although the ORs pointed in the same direction than for men (OR 0.82, 95 % CI 0.67–1.00). To further examine whether our non-significant results were observed due to a relatively small sample size we have performed identical analysis but grouping together data from previous SHARE waves (1–2, 2–4, 4–5), which indeed confirmed that gender differences remained insignificant among older adults (see Online Supplementary Table 3).

**Table 2:** Odds ratios (OR) of smoking cessation between 2011 and 2013 the SHARE sample men and women aged 50 and over. *Source* Own estimation based on SHARE, waves 4 and 5

	Men (*n* = 3345)		Women (*n* = 3115)	
	OR	CI 95 %	OR	CI 95 %
Marital transitions				
Stayed in a union (ref.)				
Became widowed or divorced	**0.359**	(0.137–0.937)	**0.458**	(0.213–0.987)
Not in a union	**0.718**	(0.570–0.904)	0.819	(0.668–1.004)
Employment transitions				
Stayed (self-)employed (ref.)				
Became retired	0.677	(0.426–1.074)	0.845	(0.511–1.396)
Stayed retired	**0.723**	(0.534–0.979)	**0.728**	(0.530–0.999)
From (self-)employed to unemployed	0.624	(0.257–1.516)	0.507	(0.178-1.446)
Sick/dis or other unemployed	**0.582**	(0.376–0.900)	1.053	(0.682–1.626)
Stayed Homemaker			0.786	(0.507–1.219)
Others	0.885	(0.628–1.248)	1.014	(0.732–1.405)

All of the models are controlled by age, education, disease incidence, and country of residence. The results of the covariates are presented in Online Supplementary Tables 1 and 2

“ref.” indicates reference categories

The values that are significantly different from 1 (at a 95 % confidence interval) are in bold

### The effect of employment losses

The transitions from employed to unemployed or retirement were not statistically significantly associated with smoking cessation for any of the two genders, although the ORs were above 1. Remaining unemployed (category “sick disabled or other unemployed”) was significantly associated with lower ORs among men (OR 0.58, 95 % CI 0.68–0.90) but not among women (OR 1.05, 95 % CI 0.68–1.63). Stayed retired had a significant negative association with smoking cessation among both men (OR 0.72, 95 % CI 0.53–0.98) and women (OR 0.73, 95 % CI 0.53–1.00) as compared to those individuals who remained employed throughout the analysed period. All other categories of the variable employment transitions were not significant, and only those for the categories “Sick/disabled or other unemployed” and “others” were (slightly) above 1 among women. Again these insignificant effects seem not to be the result of the sample size (see Online Supplementary Table 3).

### The effect of gender

The association between gender and smoking cessation was not statistically significant (OR 1.08, 95 % CI 0.94–1.24) (Table 3). The results of the interaction terms were not statistically significant at 95 % confidence level, but the interaction between gender and the category “Sick/disabled or other unemployed” was statistically significant at 90 % confidence level (OR 1.80, 95 % CI 0.99–3.27), suggesting women from this category have higher risks of smoking cessation, as compared to men.

**Table 3:** Odds ratios (OR) of smoking cessation between 2011 and 2013 in the SHARE sample men and women aged 50 and over: gender effects. *Source* Own estimation based on SHARE, waves 4 and 5

	Mod1		Mod2		Mod3	
	OR	CI 95 %	OR	CI 95 %	OR	CI 95 %
Marital transitions						
Stayed in a union (ref.)						
Became widowed or divorced	**0.425**	(0.234–0.771)	**0.381**	(0.147–0.985)	**0.426**	(0.235–0.773)
Not in a union	**0.765**	(0.659–0.888)	**0.712**	(0.567–0.893)	**0.777**	(0.669–0.903)
Employment transitions						
Stayed (self-)employed (ref.)						
Became retired	0.744	(0.530–1.043)	0.746	(0.532–1.047)	0.680	(0.432–1.069)
Stayed retired	**0.729**	(0.587–0.906)	**0.732**	(0.589–0.910)	**0.751**	(0.578–0.974)
From (self-)employed to unemployed	0.571	(0.291–1.122)	0.572	(0.291–1.123)	0.617	(0.255–1.494)
Sick/dis or other unemployed	0.764	(0.563–1.037)	0.770	(0.567–1.045)	0.584	(0.381–0.895)
Stayed Homemaker	0.735	(0.493–1.096)	0.747	(0.500–1.117)	0.749	(0.494–1.135)
Others	0.935	(0.741–1.181)	0.732	(0.589–0.910)	0.751	(0.578–0.974)
Gender						
Men (ref.)						
Women	1.079	(0.939–1.240)	1.036	(0.876–1.224)	1.051	(0.810–1.362)
Women * became widowed or divorced			1.223	(0.362–4.134)		
Women * Not in a union			1.138	(0.842–1.538)		
Women * Became retired					1.230	(0.631–2.397)
Women * Stayed retired					0.932	(0.674–1.288)
Women * From (self-)employed to unemployed					0.832	(0.211–3.278)
Women * Sick/dis or other unemployed					1.801	(0.991–3.273)
Women * Stayed Homemaker						
Women * Others					1.108	(0.706–1.737)

All of the models are controlled by age, education, disease incidence, and country of residence

“ref.” indicates reference categories

The values that are significantly different from 1 (at a 95 % confidence interval) are in bold

## Discussion

### Summary of results

Our analysis of a sample of European adults aged 50 and over indicated that marital and employment losses had different associations with smoking cessation. Over a two-year follow-up period, our results showed that transitions from being married to being divorced or widowed were significantly associated with lower chances of quitting smoking. Our findings further indicated that transitions to being unemployed or retired were not significantly associated with smoking cessation. We observed no statistically significant differences in these associations between men and women.

### Comparison of results

#### Marital losses

Previous studies on the effects of marital transitions on smoking cessation specifically among older adults are scarce. Because age seems to be an essential factor when examining the effects of marital losses on smoking cessation (Grotvedt and Stavem 2005; Jarvis et al. 2013; Umberson et al. 2008; Whitson et al. 2006) we first compare our results with the findings of studies that analysed similar age groups.

We found a negative association between becoming widowed or divorced and smoking cessation in both men and women aged 50 and over. Our results for women seem to be in line with those of a previous study conducted among middle-aged and older women in the USA (Lee et al. 2005), which might indicate that our findings for women may be generalizable for high-income countries. However, our results for men seem to contradict the findings of a US study that showed that changes in smoking behaviour were non-significant in a sample of middle-aged and older US male health professionals (Eng et al. 2005). These different results could be explained by the different outcome measures of the two studies: i.e. changes in smoking behaviour versus smoking cessation.

Comparing our results to previous findings for adult populations (Nystedt 2006), the negative association between marital loss and smoking cessation is consistent and seems to persist throughout the adult life course. Our finding of a non-significant gender difference in this association is also consistent with those of previous research that investigated the association between marital transitions and smoking cessation (Nystedt 2006) or the association between interpersonal loss (including death of the spouse) and smoking cessation (McKee et al. 2003) at adult ages.

#### Employment losses

In terms of employment losses, the non-significant differences in smoking cessation we observed between individuals who did or did not become unemployed are similar to those found in an earlier study on the effects of involuntary job loss on smoking cessation among older workers (Falba et al. 2005). Likewise, insignificant results were found in a sample of adult Germans (Schunck and Rogge 2012).

Previous studies that looked at the effects of the transition to retirement on smoking cessation, reported mixed results: one UK study reported a positive effect (Lang et al. 2007) while another, from the USA, found no significant effect (Midanik et al. 1995). Our finding of a non-significant association between entering retirement and smoking cessation among both men and women, which was derived from a large European sample, shows that the association is predominantly not significant.

Our finding that gender differences in terms of employment transitions were non-significant cannot be compared to the results of previous studies, because of the absence of gender-specific studies. It should be noted that although we do not see an effect of gender at 95 % confidence level, for “sick/dis or other unemployed” we do find an effect of gender at 90 % confidence level. This observation is important because previous studies that did not examine gender differences found that unemployed adults had lower chances of smoking cessation than employed adults (De Vogli and Santinello 2005; Giordano and Lindstrom 2011; Kriegbaum et al. 2011). It appears that these results were largely driven by men. The above example indicates that gender-specific analysis can be essential for understanding the mechanisms that underlie the aggregated results of some employment transitions.

### Explanation of results

Marital losses seem to have had important effects on smoking cessation for both men and women, and at all adult ages. At older ages the effects of marital losses were larger and more robust than the effects of employment losses. This finding suggests that among both men and women the lack of a spouse was a greater handicap for smoking cessation than the lack of employment.

Our results also showed that the associations between marital and employment losses and smoking cessation did not differ by gender. Therefore, the hypothesised underlying mechanisms of gender differences mentioned in the introduction (stress arising from different types of events combined with differences in coping mechanisms, and marriage having stronger protective effects on unhealthy behaviours among men than among women) were not sufficiently strong among older adults to have resulted in gender differences in smoking cessation. This finding seems to be in line with a previous result showing that gender differences in the protective effect of marriage for mortality did not exist at old ages (Manzoli et al. 2007). Thus, some mechanisms related to gender differences at all adult ages do not seem to apply at older ages.

The observation that our results are more in line with previous results for adult ages is quite unexpected. Previous research reported that smoking cessation rates were higher among older than younger adults (Jarvis et al. 2013). Similarly, we found that the variable age was positively associated with smoking cessation (Table 3). However, our results also showed that marital and employment losses had similar influences on smoking cessation, regardless of the age group analysed. It may be the case that that the effects on quitting smoking of the stress that accompanied these transitions were similar across individuals of all adult ages.

### Evaluation of data and methods

In this study we used European data for 13 countries to examine potential determinants of smoking cessation among older adults. So far, relatively little research has been carried out on the topic of smoking cessation among older workers and retirees, and to our knowledge this is the first study that has examined this issue for the European context as a whole.

Our study has some limitations. First, we were not able to establish causality between transitions in marital or employment status and smoking cessation. However, it is highly probable that marital and employment losses have an impact on smoking cessation via psychosocial stress (De Vogli and Santinello 2005; Falba et al. 2005; Johnson and Wu 2002; McKee et al. 2003), and it seems unlikely that these effects go in the opposite direction. Second, we used data from two periods of time (2011 and 2013), assuming that no more than one transition had occurred in terms of marriage and employment. Although several marital and employment transitions may have occurred, it is unlikely that individuals would have experienced multiple marriage and employment transitions within the relatively short two-year period. Third, our results may be influenced by other, stressful, events in life with an effect on smoking cessation. However, because we selected a relatively short period of time between the waves, the chances of this are lower compared to a longer period of time between the waves. Finally, due to data limitations we could not control for other factors that have been shown to contribute to smoking cessation, such as the presence of smokers in the household (Chandola et al. 2004), the number of cigarettes smoked per day and the onset of smoking behaviour (Broms et al. 2004). Because our results for marital and employment transitions changed only a little when we included variables that are known to be strongly associated with smoking cessation (i.e. the incidence of various diseases), we expect that our results will be proven to be robust when other, less important variables are included.

## Conclusion

In conclusion, our results indicate that in high-income countries the loss of a spouse has clear negative effects on smoking cessation not only at adult ages, but also specifically among adults aged 50 and over. Negative effects of transitions to unemployment or retirement on smoking cessation could not be demonstrated; a finding that is partly in line with the results at all adult ages. The absence of statistically significant gender differences in the observed associations for marital and employment losses seems to indicate that among adults aged 50 and over, the hypothesised underlying mechanisms differ little by gender. Health interventions should take into account the finding that the groups with lower chances of quitting smoking are affected by marital losses, irrespective of gender.

## Electronic Supplementary material

Below is the link to the electronic Supplementary material.

Supplementary material 1 (DOCX 45 kb)
