# Supplementary material for: Smoking cessation among European older adults: the contributions of marital and employment transitions by gender
Source: Eur J Ageing. 2016 Oct 21;14(2):189–98. doi: 10.1007/s10433-016-0401-4 (PMC5435786; doi:10.1007/s10433-016-0401-4)
Supplement: Supplementary file 1 — Supplementary material 1 (DOCX 45 kb) [file 10433_2016_401_MOESM1_ESM.docx]

**Supplementary table 1.** Odds ratios (OR) of smoking cessation between 2011 and 2013 the SHARE sample of men aged 50 and over

|  |  | model 1 | | model 2 | | model 3 | | model 4 | |
| --- | --- | --- | --- | --- | --- | --- | --- | --- | --- |
|  |  |  |  |  |  |  |  |  |  |
|  |  | OR | CI 95% | OR | CI 95% | OR | CI 95% | OR | CI 95% |
| Marital transitions | Stayed in a union (ref.) |  |  |  |  |  |  |  |  |
|  | Became widowed or divorced | **0.369** | (0.143-0.948) | **0.375** | (0.146-0.963) | **0.371** | (0.143-0.963) | **0.359** | (0.137-0.937) |
|  | Not in a union | **0.703** | (0.561-0.880) | **0.695** | (0.555-0.870) | **0.688** | (0.548-0.865) | **0.718** | (0.570-0.904) |
|  |  |  |  |  |  |  |  |  |  |
| Employment transitions | Stayed (self-)employed (ref.) |  |  |  |  |  |  |  |  |
|  | Became retired | 0.669 | (0.426-1.049) | 0.684 | (0.436-1.074) | 0.645 | (0.409-1.018) | 0.677 | (0.426-1.074) |
|  | Stayed retired | **0.691** | (0.519-0.920) | **0.717** | (0.537-0.957) | **0.678** | (0.506-0.910) | 0.723 | (0.534-0.979) |
|  | From (self-)employed to unemployed | 0.706 | (0.295-1.690) | 0.702 | (0.292-1.685) | 0.657 | (0.272-1.587) | 0.624 | (0.257-1.516) |
|  | Sick/dis or other unemployed | **0.595** | (0.391-0.905) | **0.602** | (0.394-0.919) | **0.545** | (0.354-0.839) | 0.582 | (0.376-0.900) |
|  | Stayed Homemaker |  |  |  |  |  |  |  |  |
|  | Others | 0.901 | (0.648-1.254) | 0.908 | (0.652-1.266) | 0.839 | (0.598-1.177) | 0.885 | (0.628-1.248) |
|  |  |  |  |  |  |  |  |  |  |
|  | Age (catgorical) | **1.059** | (1.044-1.075) | **1.056** | (1.04-1.072) | **1.056** | (1.036-1.069) | **1.047** | (1.030-1.064) |
| Education (ISCED-97) |  |  |  |  |  |  |  |  |  |
|  | 0-2: Lower secondary school (ref.) |  |  |  |  |  |  |  |  |
|  | 3: Upper secondary school |  |  | 0.752 | (0.607-0.931) | 0.745 | (0.600-0.925) | 0.823 | (0.650-1.043) |
|  | 4-6: Post-secondary school |  |  | 1.114 | (0.891-1.394) | 1.101 | (0.877-1.381) | 1.215 | (0.947-1.558) |
|  |  |  |  |  |  |  |  |  |  |
| Disease incidence | Hypertension (ref. no disease) |  |  |  |  | **1.594** | (1.181-2.152) | **1.560** | (1.151-2.113) |
|  | Cholesterol (ref. no disease) |  |  |  |  | 0.983 | (0.712-1.356) | 0.939 | (0.678-1.302) |
|  | Diabetes (ref. no disease) |  |  |  |  | 1.256 | (0.803-1.966) | 1.205 | (0.766-1.895) |
|  | Lung disease (ref. no disease) |  |  |  |  | 0.897 | (0.646-1.246) | 1.249 | (0.805-1.938) |
|  | Heart attack (ref. no disease) |  |  |  |  | **2.041** | (1.431-2.910) | **2.197** | (1.536-3.142) |
|  | Cancer (ref. no disease) |  |  |  |  | **1.787** | (1.089-2.930) | **1.811** | (1.100-2.982) |
|  |  |  |  |  |  |  |  |  |  |
| Country | Austria |  |  |  |  |  |  | 1.529 | (0.953-2.451) |
|  | Belgium |  |  |  |  |  |  | 1.172 | (0.715-1.923) |
|  | Czech Republic |  |  |  |  |  |  | 1.299 | (0.812-2.078) |
|  | Denmark |  |  |  |  |  |  | 1.265 | (0.739-2.165) |
|  | Estonia |  |  |  |  |  |  | 1.182 | (0.759-1.840) |
|  | France |  | |  |  |  | |  |  |
|  | Germany |  |  |  |  |  |  | 1.559 | (0.802-3.028) |
|  | Italy |  |  |  |  |  |  | **2.223** | (1.350-3.662) |
|  | Netherlands |  |  |  |  |  |  | **1.796** | (1.026-3.142) |
|  | Slovenia |  |  |  | |  |  | 1.325 | (0.724-2.426) |
|  | Spain |  |  |  |  |  |  | **2.048** | (1.242-3.378) |
|  | Sweden |  |  |  |  |  |  | **3.786** | (1.970-7.275) |
|  | Switzerland |  |  |  |  |  |  | 1.577 | (0.968-2.569) |

“ref.” indicates reference categories.

The values that are significantly different from 1 (at a 95% confidence interval) are in bold.

Source: Own estimation based on SHARE, waves 4 and 5.

**Supplementary table 2.** Odds ratios (OR) of smoking cessation between 2011 and 2013 the SHARE sample of women aged 50 and over

|  |  | model 1 | | model 2 | | model 3 | | model 4 | |
| --- | --- | --- | --- | --- | --- | --- | --- | --- | --- |
|  |  |  |  |  |  |  |  |  |  |
|  |  | OR | CI 95% | OR | CI 95% | OR | CI 95% | OR | CI 95% |
| Marital transitions | Stayed in a union (ref.) |  |  |  |  |  |  |  |  |
|  | Became widowed or divorced | **0.474** | (0.223-1.009) | **0.476** | (0.224-1.014) | **0.468** | (0.219-0.999) | **0.458** | (0.213-0.987) |
|  | Not in a union | **0.774** | (0.635-0.944) | **0.772** | (0.633-0.941) | **0.771** | (0.631-0.941) | **0.819** | (0.668-1.004) |
|  |  |  |  |  |  |  |  |  |  |
| Employment transitions | Stayed (self-)employed (ref.) |  |  |  |  |  |  |  |  |
|  | Became retired | 0.833 | (0.510-1.36) | 0.823 | (0.504-1.346) | 0.812 | (0.496-1.331) | 0.845 | (0.511-1.396) |
|  | Stayed retired | **0.702** | (0.521-0.944) | **0.701** | (0.520-0.946) | **0.669** | (0.494-0.906) | **0.728** | (0.530-0.999) |
|  | From (self-)employed to unemployed | 0.478 | (0.169-1.352) | 0.482 | (0.170-1.367) | 0.510 | (0.18-1.448) | 0.507 | (0.178-1.446) |
|  | Sick/dis or other unemployed | 1.038 | (0.684-1.577) | 1.042 | (0.684-1.588) | 0.980 | (0.638-1.504) | 1.053 | (0.682-1.626) |
|  | Stayed Homemaker | 0.831 | (0.552-1.250) | 0.849 | (0.560-1.288) | 0.836 | (0.548-1.273) | 0.786 | (0.507-1.219) |
|  | Others | 1.018 | (0.743-1.395) | 1.026 | (0.745-1.412) | 0.987 | (0.716-1.362) | 1.014 | (0.732-1.405) |
|  |  |  |  |  |  |  |  |  |  |
|  | Age (catgorical) | **1.052** | (1.037-1.068) | **1.053** | (1.037-1.068) | **1.053** | (1.036-1.067) | **1.046** | (1.030-1.063) |
| Education (ISCED-97) |  |  |  |  |  |  |  |  |  |
|  | 0-2: Lower secondary school (ref.) |  |  |  |  |  |  |  |  |
|  | 3: Upper secondary school |  |  | 1.129 | (0.911-1.399) | 1.139 | (0.917-1.415) | 1.191 | (0.951-1.493) |
|  | 4-6: Post-secondary school |  |  | 1.028 | (0.797-1.327) | 1.046 | (0.809-1.352) | 1.077 | (0.823-1.41) |
|  |  |  |  |  |  |  |  |  |  |
| Disease incidence | Hypertension (ref. no disease) |  |  |  |  | 1.327 | (0.956-1.843) | 1.307 | (0.937-1.822) |
|  | Cholesterol (ref. no disease) |  |  |  |  | 1.050 | (0.747-1.476) | 1.046 | (0.741-1.477) |
|  | Diabetes (ref. no disease) |  |  |  |  | 1.105 | (0.662-1.844) | 1.194 | (0.713-2.001) |
|  | Lung disease (ref. no disease) |  |  |  |  | 1.159 | (0.749-1.791) | 1.161 | (0.747-1.803) |
|  | Heart attack (ref. no disease) |  |  |  |  | 1.029 | (0.608-1.743) | 1.043 | (0.611-1.780) |
|  | Cancer (ref. no disease) |  |  |  |  | **2.048** | (1.223-3.430) | **2.056** | (1.221-3.461) |
|  |  |  |  |  |  |  |  |  |  |
| Country | Austria |  |  |  |  |  |  | 1.532 | (0.785-2.989) |
|  | Belgium |  |  |  |  |  |  | 1.492 | (0.754-2.955) |
|  | Czech Republic |  |  |  |  |  |  | 1.705 | (0.887-3.277) |
|  | Denmark |  |  |  |  |  |  | **2.441** | (1.222-4.876) |
|  | Estonia |  |  |  |  |  |  | 1.895 | (0.980-3.666) |
|  | France |  | |  |  |  | | 1.953 | (0.979-3.898) |
|  | Germany |  |  |  |  |  |  | **2.537** | (1.129-5.699) |
|  | Italy |  |  |  |  |  |  | 3.555 | (1.786-7.075) |
|  | Netherlands |  |  |  |  |  |  | 1.495 | (0.719-3.106) |
|  | Slovenia |  |  |  | |  |  |  |  |
|  | Spain |  |  |  |  |  |  | **2.817** | (1.352-5.870) |
|  | Sweden |  |  |  |  |  |  | **3.362** | (1.613-7.007) |
|  | Switzerland |  |  |  |  |  |  | 1.878 | (0.948-3.718) |

“ref.” indicates reference categories.

The values that are significantly different from 1 (at a 95% confidence interval) are in bold.

Source: Own estimation based on SHARE, waves 4 and 5.

**Supplementary table 3.** Odds ratios (OR) of smoking cessation between SHARE waves (1-2, 2-4 and 4-5) of men and women aged 50 and over (n=13,690): Gender effects.

|  |  | Gender | | Gender * marital | | Gender * employment | |
| --- | --- | --- | --- | --- | --- | --- | --- |
|  |  |  |  |  |  |  |  |
|  |  | OR | CI 95% | OR | CI 95% | OR | CI 95% |
| Marital transitions | Stayed in a union (ref.) |  |  |  |  |  |  |
|  | Became widowed or divorced | **0.408** | (0.270-0.614) | **0.360** | (0.184-0.703) | **0.408** | (0.271-0.615) |
|  | Not in a union | **0.766** | (0.691-0.849) | **0.771** | (0.662-0.899) | **0.767** | (0.691-0.851) |
|  |  |  |  |  |  |  |  |
| Employment transitions | Stayed (self-)employed (ref.) |  |  |  |  |  |  |
|  | Became retired | 1.074 | (0.887-1.301) | 1.074 | (0.887-1.301) | 1.018 | (0.792-1.308) |
|  | Stayed retired | 0.891 | (0.768-1.033) | 0.891 | (0.768-1.032) | 0.874 | (0.735-1.040) |
|  | From (self-)employed to unemployed | 0.675 | (0.454-1.002) | 0.675 | (0.455-1.003) | 0.671 | (0.407-1.104) |
|  | Sick/dis or other unemployed | **0.687** | (0.554-0.853) | **0.687** | (0.554-0.852) | 0.589 | (0.444-0.782) |
|  | Stayed Homemaker | 0.895 | (0.710-1.127) | 0.894 | (0.708-1.127) | - | |
|  | Others | 1.055 | (0.909-1.224) | 1.054 | (0.908-1.224) | 1.076 | (0.873-1.325) |
| Gender |  |  |  |  |  |  |  |
|  | Men (ref.) |  |  |  |  |  |  |
|  | Women | 0.980 | (0.892-1.077) | 0.981 | (0.878-1.096) | 0.938 | (0.791-1.114) |
|  |  |  |  |  |  |  |  |
|  | Women * became widowed or divorced |  |  | 1.225 | (0.526-2.856) |  |  |
|  | Women * Not in a union |  |  | 0.988 | (0.804-1.213) |  |  |
|  |  |  |  |  |  |  |  |
|  | Women * Became retired |  |  |  |  | 1.132 | (0.779-1.646) |
|  | Women * Stayed retired |  |  |  |  | 1.042 | (0.835-1.299) |
|  | Women * From (self-)employed to unemployed |  |  |  |  | 1.004 | (0.443-2.276) |
|  | Women * Sick/dis or other unemployed |  |  |  |  | 1.454 | (0.953-2.218) |
|  | Women * Stayed Homemaker |  |  |  |  | - | |
|  | Women * Others |  |  |  |  | 0.972 | (0.732-1.292) |

All of the models are controlled by age, education, disease incidence, country of residence and waves.

“ref.” indicates reference categories.

The values that are significantly different from 1 (at a 95% confidence interval) are in bold.

Source: Own estimation based on SHARE, waves 1, 2, 4 and 5.
